# A Practical Treatise on Organic Diseases of the Uterus

**Published:** 1844-01-01

**Authors:** 


### A Practical Treatise on Organic Diseases of the Uterus. By *John C. W. Lever*, M.D. &c. Octavo, pp. 228. London, 1843. Longman & Co.

The study of the Diseases of the Uterus, and of every thing appertaining to the Obstetrical Art, now attracts very much more attention than it did twenty or thirty years ago. Within less than the short period of a *lustrum,* we have brought under the notice of our readers important works from the pens of Drs. *Ramsbotham, Lee, Churchill, Ashwell, Montgomery,* and *Davis*—not to mention some from our brethren across the Channel; and still there seems to be a demand for more. The author of the present Treatise—which is the prize essay of the London Medical Society for 1843—is already well known as the contributor of some able articles to the Guy's Hospital Reports, which have been reviewed in some recent numbers of this Journal. His experience in obstetrical cases is necessarily very extensive, as he is the assistant accoucheur, and one of the lecturers on midwifery, at that immense institution.

We shall not linger over the introductory pages, as they contain nothing that is novel or that deserves particular notice. It is to be borne in mind that the work is professedly devoted to the *Organic* Diseases of the Uterus; the *Functional* not coming within the scope of its enquiries. The former Dr. *Lever* arranges under three heads or divisions—I. Inflammation and its Consequences; II. Diseases of a Specific Nature; and—III. Malignant Diseases.

A few general remarks may be usefully premised, before we proceed to the examination of particulars.

There are certain symptoms which may be considered as common to all the Organic Diseases of the Uterus—whether these be simple, specific, or malignant in their nature. For example, the mucous discharge from the vagina is almost always more or less increased in quantity, and altered in its characters. There is some irregularity or another in the menstrual secretion; either amenorrhœa, dysmenorrhœa, or a tendency to menorrhagia, and then the intervals between each return are usually shorter than in health. Sexual connexion, or even fatiguing exercise, especially if this be on foot, brings on pain and uneasiness in the pelvis or vagina, and often a slight discharge of blood. There is a sensation of dragging at the loins and pit of the stomach, and of an uncomfortable weight about the perineum and groins. Very generally the excretion of the urine is accompanied with some trouble and inconvenience; perhaps there is a certain degree of incontinence or strangury, the desire for micturition returning very frequently, and being attended with pain and uneasiness. The rectum too is speedily implicated; the bowels are irregular in their action; and often there is a disposition to hæmorrhoids. Along with these symptoms, denoting that there is something amiss in the pelvic viscera, there is almost always a sympathetic affection of the mammæ, when the uterus is the suffering organ. Indeed, the patient sometimes complains more of the former than of the latter part, and consults the medical man for what she supposes to be some affection in her breasts. Attention to the various circumstances now mentioned will suffice to guard *him* from committing this mistake. As the uterine disease makes progress, its characteristic symptoms are too painfully conspicuous ever to be overlooked; but then let it be remembered, that it is in the first stage only that the resources of the healing art are generally of any avail. It is therefore of paramount importance that every medical practitioner be duly alive to the signs, which usually indicate incipient organic diseases of the uterus.

We now proceed to take a rapid survey of some of the more frequent and important of these diseases; and first of—

### *Acute Inflammation of the Uterus, or Metritis.*

This disease, as might be imagined, is of comparatively rare occurrence in the unimpregnated state. Its most frequent cause, under such circumstances, (according to the experience of Dr. *Lever*), is a sudden suppression of the catamenia, and the use of stimulating injections for the cure of Leucorrhea. It may be induced also by a blow, or contusion, or by any other injury of the hypogastrium.

The symptoms may be readily predicated—sharp darting pains in the pelvis, frequent and uneasy micturition, sense of weight and bearing down in the vagina, increased by walking or any exercise. The mammæ often sympathise and become swollen and tender; the stomach is usually irritable; and there is always more or less feverish disturbance of the system.

The disease is apt to be mistaken for some malady of the bladder or of the rectum. Hence the propriety of a manual examination either by the vagina or rectum, or by both, when there is any room for doubt. The uterus will probably be found to be larger and heavier than in health, and often exquisitely painful on pressure. *Metritis*, when neglected or occurring in an unhealthy constitution, may be followed by *ramollissement* of the substance of the uterus, by suppuration in its parietes, or even—although this termination is very rare—by gangrene.

We need not say much respecting the treatment, as the proper curative means are sufficiently obvious. Bleeding, general and local, calomel and opium, saline purgatives and diuretics, rest, &c., are the main remedies to be relied on. There is only one precept of Dr. *Lever's*, on which we shall offer a remark or two. He says, " If the disease has arisen from suppressed menstruation, the re-appearance of the secretion should be strenuously attended to." This observation may be interpreted as a recommendation of the use of emmenagogue medicines. Possibly Dr. L. intended that it should be. If so, the propriety of the advice is, in our judgment, very questionable. The less that this class of remedies is used in all cases where the uterus is, or has recently been, suffering, the better. When once the organ is restored to a healthy condition, and the general constitution has recovered its tone, Nature will in general re-establish the menstrual secretion, without the assistance of any direct stimulants either to the organ itself or to the adjacent parts. We are the more induced to insist upon this point, as it is still a very prevailing opinion that, whenever the catamenia are absent, we should, as a matter of course, endeavour to provoke their return by the use of emmenagogues. The absence of the accustomed discharge is regarded as the *cause* of the constitutional disorder ; whereas, in the majority of cases, is it not rather the *effect?* Improve the general health, by rectifying the alvine and urinary secretions, by regulating the diet, the exercise, and the clothing, and by keeping the mind actively and cheerfully occupied, and in ninety cases out of a hundred, the menstrual secretion will return of itself.

Dr. *Lever* has enumerated Chlorosis among the functional diseases of the uterus. We doubt much if he is right in doing so.

Chlorosis, according to our view of its pathology, is *accompanied*, but not *induced* by the absence of the catamenia; and hence it seems to us that we may, with perfect safety, keep the circumstance of the amenorrhœa almost out of consideration, in conducting its treatment. The disease is essentially a disease of the circulating fluids, and may, it is well known, exist in the male sex as well, though not so frequently, as in the female. There is a deficiency of the red globules of the blood, and an excess of its serum. If we are to trust the recent researches of M. *Andral*—and his authority, we need not say, is not lightly to be disputed—the proportion of the red globules in chlorotic blood is less by one-half, or even two-thirds, than the normal quantity. Here is the " fons et origo mali:" we have a poor watery state of the circulating fluid, which Nature finds insufficient for the purposes of healthy nutrition and secretion—at that very period of life, when these functions ought to be the most energetic. Although we could by the use of any means provoke the catamenial secretion under such circumstances, would it, think you, have any beneficial effect upon the general health ?—we judge not. Menstruation, be it remembered, in the one sex has its analogous function (although this is not periodic) in the other; and certainly no one would ever think of administering any aphrodisiac medicines to a chlorotic boy, for the cure of his green-sickness. Let but the system once have a due supply of healthy blood permeating all its parts, and stimulating all its organs, and then will the white-waxen look give way to a rosy freshness, the faded and capricious appetite to a natural relish for wholesome food, the peevish listlessness and inaction to sprightly animation and vigour, and, with these changes, the absence of the menstrual secretion will be replaced by a due and normal regularity. Such will be the effects of a judicious hygienic treatment by steel and other tonics, by exercise in the open air, and a generous diet—not forgetting *Falstaff*'s sovereign remedy for male green-sickness. "The second property," says the old rogue, "of your excellent Sherries is the warming of the blood; which, before, cold and settled, left the liver white and pale, which is the badge of pusillanimity and cowardice; but the Sherries warms it, and makes it course from the inwards to the parts extreme; it illuminateth the face, which, as a beacon, gives warning to all the rest of this little kingdom, man, to arm; and then the vital commoners and inland petty spirits muster me all to their captain, the heart; who great, and puffed up with this retinue, doth any deed of courage."

Truly, a medical dissertation might be written upon the fat knight's prescription.

But it is high time to return *à nos moutons*, although the digression, we trust, may not be altogether unprofitable.

We have said that metritis may terminate fatally in *ramollissement*, and also in suppuration, of the substance of the uterus. Both these consequences were present in the following very instructive case, the report of which does not admit of abridgment

"I was called to Jane P———, æt. 21, a dressmaker; she complained of severe pain at the lower part of the abdomen. She had been to Greenwich Fair, and after heating herself by dancing, walked home to London. The night was cold and wet; she was lightly clad, and was menstruating at the time. On the following morning she had a rigor, which lasted for half an hour; this was followed by great heat of skin, delirium, and pain in the lower part of the belly, which continued up to the time I was called. The pulse was 120 sharp; the catamenia had disappeared; she complained of pain in micturition, and at times she was delirious. She was bled to approaching deliquium; leeches followed by cataplasms were applied to the belly; calomel, antimony, and opium were given every four hours; but all were of no avail, as she became rapidly worse, and died in twenty-four hours from the period of her attack.

"Upon a post-mortem examination, the peritoneum was found to be free from inflammation with the exception of that covering the uterus, which communicated a dry sensation to the finger. The uterus was of a dark reddish-green colour, soft and lacerable, and in one spot there was a depôt of a purulent-looking fluid about the size of a kidney-bean. The ovaries were of a dark red colour, and the ovarian plexus of veins were highly congested."* 27

---

* The late Mr. *Howship* had in his collection a preparation of an uterus, in the parietes of which there was an abscess, which contained an ounce of pus. Occasionally, the abscess has broke into the cavity of the uterus, and been discharged through the vagina.

Dr. *Lever* very properly cautions his readers of the danger of an insidious form of metritis rapidly coming on in cases of imperforate hymen, when an incision has been made through the septum, and the confined catamenia have been quickly discharged : the admission of the external air into the long-occluded cavity seems to have the same injurious effects under such circumstances, as we often observe to take place in cases of large purulent collections, when incautiously opened.

So much for Inflammation of the Uterus in the unimpregnated state. We shall now mention a few memoranda respecting the disease, when it occurs during pregnancy, or—as is much more frequently the case—after delivery. As might be expected, any external violence is greatly more apt to injure the gravid than the empty womb. But, besides this cause, we should remember that all attempts to induce premature labour, either by mechanical means or by the administration of the *secale*, or other medicines, are apt, in certain constitutions, to induce inflammation of this organ. It is highly necessary, therefore, that medical men be constantly on the watch, under the circumstances now alluded to, against the insidious and often unperceived invasion of the disease.

Dr. *Lever* is of opinion that the very firm adhesion of the placenta to the womb—sometimes so great as to resist all justifiable attempts to detach it—is occasionally owing to lymph having become effused between the opposing surfaces, in consequence of a previous attack of metritis. A still more serious result of the disease in question is softening of the substance of the uterus; for then there is no little danger of its becoming ruptured, if the labour be hard and difficult. Our author mentions a curious case, where an abscess formed on the right side of the os uteri in a lady who was six months pregnant: after much suffering, the abscess broke and gradually healed up. Labour came on next month, and she was delivered of a dead child. Ultimately she quite recovered.

*Subacute Metritis* may be primary, or it may be the *sequela* of a more active attack. It is not unfrequently owing to the injudicious administration of the *secale cornutum*. "I had under my care," says Dr. *Lever*, "five females attended by the same individual, to all of whom he had given the secale, and who were affected by the same symptoms: central pains, increased upon assuming the erect posture, by defæcation, and micturition; tenderness above the pubes; thick white discharge, at times streaked with blood. On vaginal examination, the uterus was found to be enlarged, its os tumid and tender, and great pain was excited by pressing with the finger upon the cervix."

In the treatment of the subacute form of the disease, it will often be found of great utility to draw blood itself from the affected organ, either by means of leeches applied to the cervix of the uterus, or by scarifying it, as recommended by the late Mr. *Fenner*.* Cupping over the loins is also an excellent remedy, and will often supersede the methods now mentioned.

"The patient should be made to rest a great deal, and, if married,

---

* Our readers will find a description of the instrument, and means of using it, in the 64th number of the Medico-Chirurgical Review.

to 'take the widow's sombre cap' for some time, as Dr. L. delicately expresses it. The use of the hip-bath, and of anodyne injections—as the Decoct. papaver. vel Conii, to which the liquor Plumbi at first, and subsequently alum, may be added—is not to be neglected. A mild diet, as a matter of course. Our author has not had occasion, we presume, to remark the *metritic* effects of coffee, which M. *Lisfranc* has dwelt so much upon—else no doubt he would have prohibited it. But the Frenchman's opinion is not likely to have much weight either on this, or on any other, subject.

In the more chronic cases, and especially when the uterus has become enlarged and hypertrophied, the internal use of the milder preparations of mercury, also of iodine,* conium, &c., will be found necessary. The muriate of ammonia, a medicine in high favour among the German physicians, deserves to be more generally tried by ourselves than it has hitherto been. An issue in one of the groins is a very potent, although a very unwelcome, remedy. Warm salt-water bathing is an admirable restorative, when all inflammation has been subdued.

It may be remarked that women, who have suffered from hysteritis, do not often become pregnant afterwards. Whether this be owing to the closure of the uterine openings of the Fallopian tubes, is uncertain: it is certainly probable.

We close our remarks on this branch of our subject with the following sensible remarks on the importance of forming a correct diagnosis, in cases of induration of the os and cervix of the uterus.

> "Induration, the result of chronic inflammation, may be known by its regular feel, and by the history of the case. In most instances there has been the creamy discharge, or, to use Sir C. M. Clarke's words, 'a mixture of starch and water made without heat.' Induration, the commencement of malignant disease, is in nodules, the malignant depositions being generally secreted in central patches. It must not, however, be forgotten that malignant disease frequently establishes itself in the mouth or neck of a womb that has become indurated by long-continued chronic inflammation: and although we cannot say that the one is the cause of the other, yet it is very certain that, when induration is allowed to go on unheeded, or even when it is stationary in many cases (as in the mamma), malignant disease will be developed at that period of life when the catamenia take their departure." 64.

*Ulceration of the Os and Cervix Uteri.*

This is either of a simple, of a specific, or of a malignant character. The *first* of these forms is generally preceded by symptoms of inflammation—shiverings and febrile re-action, a dull pain in the pelvis and a sense of dragging in the loins, and of weight about the fundament. All these symptoms are usually aggravated at each menstrual period. Often there is a most distressing pruriginous itching of the external pudenda, and very generally more or less leucorrhœal discharge, which is sometimes tinged with blood, especially after sexual connexion, or a manual examination. The size of the ulcerated spots may vary from that of a pin's

---

* Dr. *Lever* is partial to the compound of these two potent remedies—the protiodide of mercury.

head to a shilling, or thereabouts. To judge aright, it is necessary to examine them by means of a speculum: Dr. *Lever* uses one of glass, blown for the purpose, stout, and larger at one end than the other. In some cases, the ulcer is quite superficial and looks like a mere abrasion of the mucous lining; in others, it resembles a cut surface; while in others still, its edges are irregular and jagged, and the surface looks angry and irritable. The pressure of the finger will often cause it to bleed.

With respect to the *treatment* of simple ulceration of the os and cervix uteri, it will generally be found very useful to abstract a little blood locally, either by means of cupping over the sacrum, or leeches to the groins and vulva, or to the uterus itself. The tepid hip-bath every, or every second, night, and emollient anodyne injections, are useful adjuvants. Saline aperients and diaphoretics during the day, and mild sedatives at bed time, are not to be neglected. When all the inflammatory symptoms are subdued, the ulcerated spots should then be touched with the nitrate of silver, applied in substance by means of the speculum, every third or fourth day; and the general health strengthened by the use of tonics, sarsaparilla, a mild yet generous diet, and a residence, if possible, at the sea-side for some time. Sexual intercourse should, as a matter of course, be avoided.

When a prolapsus of the uterus remains for some time unreturned, the os and cervix of the organ often become the seat of troublesome ulcerations. When these are indolent and irritable, an excellent application is a poultice made with crumbs of bread and a lotion, composed of black wash and extract. Opii.

*Venereal Ulcerations* are, as might be expected, much more troublesome and difficult of management than those which we have been describing. They exhibit the common characters of genuine chancres; having hard, elevated and ragged edges, and their surface covered with a gray, sanious layer of discharge. It is unnecessary to say more on this subject; or to enlarge on the mode of treatment; as this must be conducted on exactly the same principles as when the disease is situated elsewhere. The *lotio nigra,* used warm, is an excellent application at first; and Plummer's pill, the hydriodate of potash, and sarsaparilla are the best internal remedies.

*Gonorrhœa* in the female is often accompanied with the existence of superficial ulcerations and herpetic eruptions about the os tincæ, which is then usually affected with an erythematic inflammation: the mucous glands too around the os and cervix are generally swollen and enlarged. For the relief of these symptoms, the local detraction of blood by cupping or leeches, and the use of cooling medicines, are most proper at first. Afterwards an injection of the nitrate of silver—commencing with 3 grains to an ounce of water—will, if properly employed, generally serve to remove all the symptoms.

### *Enlargement of the Glands of the Os Uteri, giving rise to Prurigo Pudendi.*

In some cases, our author remarks, after inflammation of the os and cervix uteri has ceased, patients complain of an intolerable pruritus in the vagina and external parts—just as there is apt to be an itching of the glans penis in affections of the prostate gland and urinary bladder. The complaint is often most unmanageable, and resists all the usual means of allaying irritation. Nothing abnormal can be seen, or detected by examination with the finger; but, if the speculum be employed, we may perhaps discover upon the os uteri numerous small granules, not unlike millet-seeds, white and soft, and seemingly vesicular.

In treating this troublesome complaint, it may be necessary to have recourse to local bleeding by cupping or leeches in the first instance; but, if there be no inflammatory tenderness present, we should at once have recourse to injections containing the nitrate of silver, (gr. v.—x. to an ounce of water,) or the sulphate of iron, (ʒj. to the pint): these may be used two or three times daily.

In scrofulous habits, tubercles, containing a cheesy matter, are sometimes developed in various parts of the uterus and its appendages. These may be of a globular shape, isolated and detached from each other; but the most common form of the disease consists in the deposit of tuberculous matter in the exterior or interior surface of the uterus, simulating other organic diseases of this organ, and often causing great perplexity in the diagnosis. Dr. *Lever* relates a very interesting case, which we give considerably abridged.

A young lady, whose health previously had been excellent, was suddenly seized, when coming down stairs, only ten days after marriage, with a pain in the hypogastrium, accompanied with sickness. She missed her next monthly period, and the sickness and tenderness of the abdomen gradually increased. A week or so afterwards, there was a decided fulness in the hypogastric region, and on careful examination a tumor could be felt there. The symptoms became worse and worse. The case was regarded as one of tubercular peritonitis: the tumor in the region of the uterus might be a large tubercle, or a cluster of tubercles. On examination per vaginam, the uterus was found to be completely fixed and immoveable. Four months and a half after the first seizure, the patient died from a rupture of the intestines. On dissection, the whole of the peritoneum was found to be studded with tubercles. Attached to the uterus was one of very large size; and, in addition, the entire substance of the organ was discovered to be infiltrated with a deposition of tuberculous matter.

*Corroding Ulcer of the Uterus* is a much more serious disease than any yet alluded to. The ulceration seems to have a good deal of the *lupus* character, corroding or eating away the parts affected, but not accompanied—as Sir *Charles Clarke* observes—with any hardness, thickening, or deposit of new morbid matter, either in the uterus itself or in the adjacent parts.

"When a uterus," says Dr. Lever, "affected with this disease is examined after death, it will be found to have a greater or less portion removed by ulceration, which extends itself in a circular manner, so as to completely destroy the cervix and part of the body, a small quantity of cellular tissue alone connecting it with the vagina. Sometimes it is found that the disease has attacked the anterior or posterior surface of the organ only, spreading from thence to the bladder or rectum. Sometimes the ulceration extends to the fundus uteri, and destroys the greater part." 146.

The disease seems to commence in the submucous glandular structure about the cervix; for it generally appears first in this part, and thence extends downwards and upwards. It occurs most frequently about "the change of life." The origin and progress of this destructive form of uterine ulceration are unfortunately most insidious; and often the disease has existed for a considerable time, and produced great mischief, before its presence has been even suspected. There may, indeed, have been occasional pain and uneasiness in the pelvis, a sensation of heat, leucorrhœa, &c. experienced for some time; but no particular notice may be taken of these symptoms: and the apprehensions of the patient are often first awakened by an unexpected occurrence of hæmorrhage. On now making an examination, we shall probably be surprised at the extent to which the mischief has already proceeded. Usually the patient does not complain of pain, even when the finger is passed over the ulceration; she merely expresses herself as feeling sore. The hæmorrhage, which may have first drawn attention to the disease, will probably continue to recur at intervals; and between these there is usually a discharge of offensive sanious matter; very rarely this is of a proper purulent character. We need scarcely remark, that, ere long, the constitutional health is very sensibly impaired; all the symptoms of feverish hectic decay supervening. The degree of local suffering varies very much in different instances; in some, it is very severe, while in others scarce any is experienced. Hæmorrhage or diarrhœa frequently accelerates the fatal issue; occasionally peritonitis supervenes, and rapidly exhausts the powers of life.

The essentially distinctive character between the Corroding ulcer of the uterus and Carcinomatous ulceration of this organ is, that in the former there is a mere destruction of the affected part, without any formation of new morbid matter in its place; whereas, in the latter, there is always an extensive deposition of an heterologous formation into the surrounding cellular tissue, as well as into the substance of the uterus itself—by which this organ is rendered more or less immoveable within the pelvis, in consequence of its increased bulk and weight, as well as by the adhesions that have been formed all around. In the Corroding ulcer, on the other hand, the womb (or as much of it as may be left,) may be freely moved about; perhaps even to a greater extent than in health, on account of the removal of some portion of the pelvic contents by ulceration affording more space.

What shall we say of the treatment of this formidable complaint? Can we do more than merely mitigate the sufferings of the patient, when it is once fairly established?—and, alas! this is too frequently the case when the medical man is first consulted. We fear not. Dr. *Lever* is in the habit of applying the nitrate of silver, either in substance or in solution; but no encouragement is given by him of any permanent good being effected. As a matter of course, much relief will often be afforded by the frequent use of soothing anodyne injections; none is better than tepid milk and water, to which syrup of poppies or the liquor opii is added.

If ever any means of decidedly arresting or curing this formidable disease be discovered—and we see no good reason to despair of some success —it will, we predict, be by the internal administration of some remedy that exercises a peculiar effect on the capillary vessels and their contents. The Oxymuriate of Potash has recently been tried with excellent—at least temporarily—effects in many cases of Lupous ulceration of the face; and why may it not produce similar results on the womb, when affected in a somewhat like manner? Then there is the Muriate of Ammonia, to which we formerly alluded; the Liquor Potassæ—a truly valuable medicine in many inveterate complaints of the internal viscera as well as of the skin—the preparations of Arsenic, Iron, &c. There is also one very common medicine, which is, we think, far too much overlooked in the present day, although it has certainly very soothing effects in a variety of painful diseases, independently of its salutary effects as a mild aperient—we mean Sulphur. As it seems to pervade every part of the living frame, it may reasonably be supposed to exercise a considerable effect upon the extreme vessels and nerves. Often have we occasion to observe its singularly quieting effects in numerous neuralgic and other maladies.

We proceed now to make a few observations on that most sad and sorrowful of diseases, *Carcinoma of the Uterus.* Under this general term, Dr. *Lever* includes those heterologous formations, whether of an Encephaloid, Scirrhoid, or Colloid form, which have been differently named by the various writers, who have described their pathology. All these varieties of the disease seem to be constitutional—or originating in a cachexia of the general system—either from the very first of their appearance, or very shortly after they have been once developed. The affected tissues become gradually more or less disorganised, losing entirely their normal and original structure, and degenerating into the morbid growth, to which we give the name of Cancer. According to *Muller*, its germinal cells are formed from a real "seminium morbi," which develops itself between the tissues of the affected organ. As we remarked above, there is always a deposition, to a greater or less extent, of the morbid matter into the surrounding cellular tissue, so that the diseased parts become matted and agglutinated together, and thereby are much less moveable than in a state of health. We shall not at present enter into any description of the anatomical characters of the various morbid formations, that are comprehended under the term of Carcinoma.

Unfortunately our knowledge on this point has not, as yet, been of any avail in suggesting a useful remedy against this truly dreadful disease.

According to the experience of our author, it is of much more frequent occurrence than is generally imagined; the registers of the obstetric patients of Guy's Hospital shewing that the proportion of cases of Carcinoma Uteri to those of other (organic, we presume) uterine diseases is not less than as 1 in 7, or 13·5 per cent. In a paper published in the Transactions of the Medico-Chirurgical Society for 1839, Dr. *Lever* has shewn that a large proportion of carcinomatous patients will be found to have suffered from uterine derangements—most frequently dysmenorrhœa—in their previous life. His subsequent observations confirm the correctness of this remark.

The early symptoms of the disease are usually indistinct and unsatisfactory; it is only after a careful examination per vaginam has been made, that the existence of the real evil is ascertained. In the early stage, the os uteri is generally hard, irregular, perhaps fissured, and projecting lower down than usual. On examination, the finger feels as if there were a number of grains of shot or gravel under the mucous membrane of the os and cervix: these are the enlarged and indurated muciparous glands. When pressure is made firmly upon these, the patient often experiences a dull pain and sense of sickness. If a speculum is used, the os tincæ will usually be seen to be of a deep crimson colour; but the projecting shot-like points have a bluish hue. As yet, the disease appears to be confined to the orifice and neck of the womb, and the organ is still moveable as in health. Fortunately this stage will sometimes last for several years, if nothing occur to aggravate the morbid action.

The second stage, or that of Confirmed Scirrhus, is indicated by the augmented size of the granular or shot-like bodies, giving to the os tincæ an irregular and knobby feel; by a gradual descent lower and lower of the uterus; and by an increase of the pain and local uneasines; the discharge, although greater in quantity than before, is not yet fætid or at all sanious. On examination with the finger, the neck and body of the womb are found tumefied and hard; the os tincæ is more open than it was before; its lips are rigid, and usually notched in two or three places, but without any breach of surface. The organ is now felt to be less freely moveable than it was in the first stage; but it does not become fixed in the pelvis, till the commencement of the third.

"Just previous to ulceration taking place, if careful examination be made, we shall find that some part of the tumid and indurated viscus is softer than the rest; at this spot ulceration will occur, and considerable pain will be caused when the examining finger makes pressure." 178.

As might be expected, the general health of the patient now suffers more deeply than before; her sleep is disturbed, her appetite fails, and she gradually becomes weaker and weaker. Urticaria is a not unfrequent complication, and often causes great additional distress.

The *third* stage is characterised by the occurrence of ulceration in the diseased part. As in open cancer elsewhere, all the sufferings are now tenfold more severe. The pains are described as lancinating, or stabbing, or burning, or gnawing; or perhaps all these horrid feelings are, as it were, blended together: they shoot from the womb to the pubes, and backwards to the anus and down the thighs. Not unfrequently there is a great deal of neuralgic pain, along the course of the sciatic or crural nerve felt at the same time: cases have even been known where by far the most severe pain was situated in the foot, and but very little felt in the uterine region. One or both of the lower extremities often become œdematous. Usually the first indication of ulceration having commenced, is a sudden loss of blood: this is sometimes very considerable. When it ceases, the discharge is usually found to be fætid, and of a darker colour than it was before. From its acrimony, the orifices of the vagina and anus are apt to become fretted and excoriated. There is always more or less dysuria, at this period of the disease. The rectum also sympathises; and thus every action of the bowels and bladder becomes a cause of increased suffering and distress. In some cases the ulceration extends either to the bladder or rectum; and then a fistulous communication is formed between the vagina and one of these viscera.

"Examination 'per vaginam' enables us to detect a hard irregular immovable mass, filling the pelvis, and about its centre the os uteri, which is more open than natural, its edges being thickened and ulcerated, the ulceration may extend completely round the cervix, or the anterior or posterior half alone may be affected and extend ultimately to the bladder or rectum; the ulceration is generally tender upon pressure, and the examining finger when withdrawn is covered with fetid sanies, frequently tinged with blood." 182.

It is unnecessary to pursue this melancholy picture; perhaps in the long catalogue of human ills, there is none more hard to bear, or more melancholy to witness, than cancer of the uterus. To talk of curing this disease, when ulceration has once commenced, is vain and foolish; nay, it is worse; it is wicked and cruel; for it is a wilful lie.

It is gratifying, however, to find that many of the best authorities of the day agree in holding out a reasonable prospect of success from judicious medical treatment, if the disease be attacked in its incipient stage. After the inflammatory and congestive symptoms have been relieved by local detraction of blood, the system should be slowly brought under the influence of Mercury, by the continued use of small doses of its milder preparations: the inunction of the blue ointment may be used at the same time. Afterwards, the use of Iodine, Arsenic, &c. in some bitter infusion, will tend to benefit the general health, while these medicines often serve to exercise a beneficial effect on the local disease. The establishment too of a constant discharge from some part in the neighbourhood of the disease, as by an issue over the sacrum or in one of the groins, has unquestionably been of great utility in some cases. We need scarcely say, that all fatiguing exercise, and sexual intercourse, must be strictly prohibited. Mild anodynes at bed-time are generally advisable.

It is wholly unnecessary to repeat even the mere names of the host of medicines that have been recommended, at different times, for the relief of this disease in its advanced stages. Suffice it to say that, according to the experience of Dr. *Lever*, Arsenic has unquestionably often no inconsiderable power in lessening severe pains, if not in controlling the morbid action. He reports also favourably of the effects of Iodine, at least before ulceration has taken place. As a matter of course, nothing can be done without having recourse to narcotics—administered internally, in the form of injection or of suppository, and used externally as an embrocation. These must be every now and then altered, to suit the varying condition of the patient.

We shall not now discuss the propriety of ever having recourse to a surgical operation for the removal of a Cancerous Uterus. Although excision is reported to have succeeded in a few cases, it has ever appeared to us to be all but unjustifiable. Better, we think, let the patient sink under the sufferings which Nature herself has appointed, than for the medical man to incur the almost inevitable risk of rapidly accelerating her death. Of nineteen patients alluded to by Dr. *Lever*, 16 died, we are told, *in consequence of the operation.* Surgeons are becoming every year more chary of extirpating even a cancerous mamma; for, alas! in the majority of cases, the disease re-appears either in the original locality, or elsewhere. How much greater is the danger, when the uterus is the affected organ! True, we not unfrequently read of cases in the French Journals, where excision of the os and part of the cervix uteri was performed, it is said, with brilliant success; but we have every reason to believe that often there was no malignant disease present at all, and that the cases,—at least many of them,—might have done perfectly well, had no operation been performed.

Dr. *Lever* has a section devoted to the consideration of "Pregnancy associated with Cancer of the Uterus." This truly melancholy compliplication is not so unfrequent as many may suppose. We need scarcely say that the disease of the womb is inevitably aggravated, if the pregnancy makes any considerable advance before delivery takes place; and that, if it does, the life of the child is usually lost in consequence of the extreme difficulty of it passing through the contracted and undilatable orifice. There is one remark of our author on the management of certain cases of this kind, which calls for some comment. He says:—

"In cases where there is so much structural change that the application of the forceps is impracticable, and where the child is indisputably alive, as proved by its movements and by auscultatory signs, it is of great importance to determine whether we are justified in opening the child's head, and destroying its life, or whether we should perform the Cæsarean section. This is a question which I think demands serious consideration. In many cases on record I am of opinion that the life of the child might have been spared, if such an operation had been had recourse to, whilst several of the mothers died during labour, or soon after delivery, and in others their miserable existence was prolonged but for a few weeks." 218.

We admit that the question proposed does demand *serious consideration;* but, in our opinion, there never ought to be a moment's hesitation as to the practice to be pursued. In no case ought the life of the mother to be rashly jeopardised, nay rather almost inevitably sacrificed, in the hope of preserving that of her child; surely there cannot be any reasonable grounds for expecting the recovery—even for a brief time—of a woman, affected with malignant disease of the uterus, after the performance of the Cæsarian operation. The fœtus may indeed be discovered to be alive; but what then?—let it be remembered that, independently of the comparative rarity of a child "ripped from its mother's womb" surviving for any length of time, the chances of its *viability* are much less in the instance now under consideration than in the other cases, where the operation is usually recommended or has been performed.

A healthy offspring can scarcely be expected from a diseased parent; and so experience has found. Not many of the children of cancerous mothers are ever fully developed, or survive long. In two out of the six cases, related by Dr. *Lever*, it would seem that the fœtus was small, and under the standard size; in a third, it was dead, (although the woman conceived subsequently and gave birth to a living seven-months' child), and in a fourth, it must have been dead for some time before delivery, for the cuticle was peeling off in several parts.

Let not therefore any British practitioner ever, for one moment, entertain the idea of performing the Cæsarian operation under the circumstances supposed, if the child can be brought away, at all safely to the mother, by embryotomy. Whether it would not be better to induce premature labour at an early period of pregnancy, when the degree of the dilatation of the os uteri, required for the passage of the fœtus through the diseased orifice of the womb, is much less considerable, is a question that we cannot enter upon at present. The history of one of Dr. *Lever's* cases (XL) might, we think, be adduced in favour of its propriety; although Dr. *Blundell*, who was consulted upon it, thought otherwise.

One or two Chapters of the present work still remain unreviewed—those on Polypi, and Fibrous Tumors of the Uterus; but we shall not touch upon them just now; as we think it better not to introduce any topic that may have the effect of withdrawing the attention of the reader from the important subject of Inflammatory and Ulcerative Disease of the Organ, in various conditions of the system.

In conclusion, it is only fair to Dr. *Lever* to say, that we have been much gratified, on the whole, with the perusal of this Essay;—sensible and judicious throughout, it is evidently the production of an experienced and discerning practitioner.

